# Outcome prioritisation tool for medication review in older patients with multimorbidity: a pilot study in general practice

**DOI:** 10.3399/bjgp17X690485

**Published:** 2017-03-28

**Authors:** Jojanneke JGT van Summeren, Jan Schuling, Flora M Haaijer-Ruskamp, Petra Denig

**Affiliations:** Department of General Practice, University of Groningen, University Medical Center Groningen, the Netherlands.; Department of General Practice, University of Groningen, University Medical Center Groningen, the Netherlands.; Drug Utilisation Studies, University of Groningen, University Medical Center Groningen, the Netherlands.; Drug Utilisation Quality, Department of Clinical Pharmacy and Pharmacology, University of Groningen, University Medical Center Groningen, the Netherlands.

**Keywords:** decision aid, general practice, medication review, multimorbidity, patient preference, polypharmacy

## Abstract

**Background:**

Several methods have been developed to conduct and support medication reviews in older persons with multimorbidity. Assessing the patient’s priorities for achieving specific health outcomes can guide the medication review process. Little is known about the impact of conducting such assessments.

**Aim:**

This pilot study aimed to determine proposed and observed medication changes when using an outcome prioritisation tool (OPT) during a medication review in general practice.

**Design and setting:**

Participants were older patients with multimorbidity (aged ≥69 years) with polypharmacy (five or more chronic medications) from the practices of 14 GPs.

**Method:**

Patients were asked to prioritise four universal health outcomes — remaining alive, maintaining independence, reducing pain, and reducing other symptoms — using an OPT. GPs used this prioritisation to review the medication and to propose and discuss medication changes with the patient. The outcomes included the proposed medication change as documented by the GP, and the observed medication change in the electronic health record at follow-up. Descriptive analyses were conducted to determine medication changes according to the prioritised health outcomes.

**Results:**

A total of 59 patients using 486 medications prioritised the four health outcomes. GPs proposed 34 changes of medication, mainly stopping, for 20 patients. At follow-up, 14 medication changes were observed for 10 patients. The stopping of medication (mostly preventive) was particularly observed in patients who prioritised ‘reducing other symptoms’ as most important.

**Conclusion:**

Using an OPT leads mainly to the stopping of medication. Medication changes appeared to be easiest for patients who prioritised ‘reducing other symptoms’ as most important.

## INTRODUCTION

With the ageing of the population, the number of people with multiple chronic conditions, or multimorbidity, is increasing.^1^^–^3 Such multimorbidity leads to polypharmacy, especially among the eldest.^2^ The situation may be exacerbated by the application of clinical guidelines, which are usually developed for the management of single diseases.^1^^,^^4^ Treatment according to the respective guidelines for patients with multimorbidity results in polypharmacy, because no attention is given to prioritising recommendations for individuals in whom the treatment burden weighs heavily.^1^

Healthcare providers, such as prescribers and pharmacists, have taken actions to reduce inappropriate polypharmacy.^5^ Methods developed to reduce inappropriate prescribing in older patients with multimorbidity include the STOPP/START criteria and the Beers criteria.^5^ Although this may reduce prescribing of medication that is considered inappropriate from a clinical point of view, it is not clear whether the individual patient’s needs and preferences are sufficiently taken into account.^6^^,^^7^ GPs have reported being hesitant to use patient priorities instead of evidence-based guidelines to deprescribe medication, and would welcome decision support when dealing with multiple guidelines for one patient.^8^

Fried *et al* developed a simple tool — the outcome prioritisation tool (OPT) — to elicit preferences of older persons based on prioritisation of four universal health outcomes: remaining alive, maintaining independence, reducing pain, and reducing other symptoms. Prioritisation of these health outcomes differed between individuals.^9^^–^11 Individual preferences can be used as a guide to treatment decisions in patients with multimorbidity with polypharmacy for whom disease-specific guidelines are no longer sufficient.^12^ The application of a tool to assess patient treatment priorities and preferences during medication review in patients with multimorbidity in practice has not yet been tested.^7^

The aim of this study was to determine proposed and observed medication changes when using an OPT during a medication review in older patients with multimorbidity with polypharmacy. A secondary aim was to explore the relationship between the prioritised health outcome of patients and the type of medication change, such as a stop, a dose adjustment, or a switch.

## METHOD

### Study design and participants

In this pilot study, each GP used the OPT to conduct a medication review for between two and seven patients. GPs, who were mostly also GP trainers, working in the northern Netherlands were recruited.

Patient inclusion criteria were ≥69 years of age, two or more chronic diseases (one of which had to be cardiovascular disease), and daily use of five or more medications. Patient exclusion criteria were cognitive impairment, a life expectancy of <6 months, and insufficient understanding of the Dutch language, all based on the judgement of the GP. Patients provided written informed consent.

**How this fits in**

Several methods have been developed to conduct and support medication review in older persons with multimorbidity. However, this is the first study in which health outcome prioritisation is used during medication review of older multimorbid patients by GPs. In contrast to medication reviews conducted by community pharmacists, this study showed relatively more proposed medication stops and dose decreases, and fewer switches and additions of new medications. Stopping medication appeared to be easier for patients who prioritised ‘reducing other symptoms’ as the most important health outcome, compared with patients who prioritised ‘remaining alive’ or ‘maintaining independence’ as the most important. Further research is needed to determine whether patients benefit from a medication review with an outcome prioritisation tool.

### Medication review using the OPT

The intervention took place from October 2013 to April 2014. GPs were instructed about the use of the OPT to elicit patients’ prioritisation (for example, www.optool.nl). The OPT is a conversation support tool with four movable buttons sliding on a scale of 0 to 100, each representing one of the four health outcomes.^9^ When using the OPT in a consultation or home visit, the GP first explained the four health outcomes. Patients then had to score the four health outcomes on their importance, prioritising between the outcomes. Next, the GP reviewed the medication in view of the patient’s prioritisation. No specific guidance was provided, leaving the GPs free to make their own decisions to propose medication changes. For example, if a patient prioritised reducing pain as most important and remaining alive as less important, the GP might propose to maintain medication for symptom relief but stop preventive medication. When discussing this with the patient, attention was to be paid to the possible consequences of medication changes on other health outcomes, such as maintaining independence. The GP could decide to plan a second consultation, for example, to allow consultation with a pharmacist or to give the patient more time to reflect on the OPT score. If desired, a relative or friend could accompany and support the patient during the consultation.

### Outcome measure

The outcome measure consisted of the proposed medication change as documented by the GP, and the observed medication change in the electronic health record (EHR) at follow-up.

### Data collection and analysis

All medication used by the patient before the intervention, including dosage and regimen, was recorded by the GP on a structured questionnaire. Next, the patient’s scores on the four health outcomes (OPT scores) were recorded by the GP. Having compared the patient’s OPT score to the patient’s medication list, the GP recorded the proposed medication changes for adapting the treatment to the patient’s prioritised health outcome on the questionnaire. The observed medication changes at follow-up were retrieved from the EHR of the patient at least 2 months and not later than 8 months after medication review. In the Netherlands, every GP uses an EHR software package approved by the Dutch College of General Practitioners. In this system, the patient’s prescriptions and prescription changes are recorded by the GP. New and changed prescriptions are directly sent digitally to the patient’s pharmacist. In this study all chronic prescriptions (duration >3 months) were included, whereas non-prescription medication, such as paracetamol, eye/ear drops, and dermatologicals, were excluded. The type of medication change was categorised as being a start, a stop, a dose increase, a dose decrease, or a switch. The patient’s sex and age were retrieved from the EHR, and education and living conditions were collected using a questionnaire.

Descriptive statistics were used to describe the population, as well as the proposed and observed medication changes. Medications were classified using the therapeutic groups of the anatomical therapeutic subgroup (ATC) system (http://www.whocc.no/atc/structure_and_principles).

## RESULTS

### Characteristics of GPs and patients

In total, 17 GPs and 63 patients were recruited. Three GPs dropped out because of time constraints. The median work experience of the 14 participating GPs was 28 years (interquartile range [IQR] 12.5–33.0 years); six were females; nine worked in a city; five were dispensing GPs; three worked in a single practice, two worked in duo practices, and nine worked in seven group practices. The questionnaire was lost in the mail for four patients (resulting in all questionnaires lost for one GP), and two additional patients were lost to follow-up in the EHR. This resulted in 59 patients for the analysis of proposed medication changes, and 57 patients for the analysis of observed medication changes. The patients’ median age was 83 years (IQR 81–86 years), 30 were female, and patients’ used on average 9.4 ± 3.1 medications. Living conditions were unknown for two of the patients, six patients lived in a home for older persons, and 52 patients lived independently.

### Proposed and observed medication changes

Before the medication review, the 59 patients used a total of 486 medications (Table 1). Looking at the most frequently prescribed therapeutic subgroups, 57 antithrombotic agents (B01) were prescribed for 49 patients, 42 diuretics (C03) for 33 patients, 41 agents acting on the renin–angiotensin system (C09) for 40 patients, and 41 antacids (A02) for 40 patients.

**Table 1.** Number of medications per therapeutic subgroup prescribed before the consultation, and number of proposed and observed medication changes in 59 patients

**Medication therapeutic subgroup (ATC code)**	**Medications used before, consultation, *n***	**Proposed medication changes after consultation, *n***	**Observed medication changes at follow-up,^a^*n***
Lipid-modifying drugs (C10)	32	7	2
Analgesics (N02)	12	2	1
Urologicals (G04)	14	2	0
Laxatives (A06)	16	2	2
Mineral supplements (A12)	10	1	1
Psycholeptics (N05)	15	1	0
Respiratory drugs (R03)	32	2	0
Antacids (A02)	41	2	0
Diabetes drugs (A10)	27	1	1
Antithrombotics (B01)	57	2	1
Beta-blocking drugs (C07)	34	1	1
Diuretics (C03)	42	1	0
RAS-inhibitors (C09)	41	0	0
Cardiac drugs (C01)	18	0	0
Ca-channel blockers (C08)	14	0	0
Others	81	10	5
**Total:**	**486**	**34^b^**	**14^c^**

a Follow-up data were available from 57 of the 59 patients.

b Proposed changes were recorded in 20 patients.

c *Observed changes were recorded in 10 patients. ATC* = *anatomical therapeutic subgroup. Ca* = *calcium. RAS* = *renin–angiotensin system.*

In total, 34 changes in medication (7% of 486) were proposed for 20 patients (34% of 59) (Table 1). The most common therapeutic subgroups with relatively the most proposed medication changes were lipid-modifying drugs (C10), analgesics (N02), urologicals (G04), laxatives (A06), and mineral supplements (A12). In three of the 15 most frequently prescribed therapeutic subgroups no changes were proposed — agents acting on the renin–angiotensin system (C09), cardiac drugs (C01), and calcium channel blockers (C08). The types of proposed changes were mainly stopping the medication (22 times) or decreasing the dosage (six times). No new medication was started, but three switches to another medication from the same therapeutic subgroup were proposed. For two medications, an increase in dosage was proposed.

Following up on the 34 proposed medication changes in 20 patients, the authors observed 14 changes in the EHR of 10 patients (Table 1). This included 13 medications being stopped and one medication with a decrease in dosage (Table 2). In four of these cases, medication was stopped after a proposed decrease or switch. The observed changes occurred in a wide range of therapeutic subgroups, but no changes were observed for the proposed changes of drugs used in benign prostatic hypertrophy (G04, urologicals), hypnotics/sedatives (N05, psycholeptics), and bronchodilators (R03, respiratory drugs) (Table 1). Proposed medication changes that were not observed in the EHR occurred for all types of changes.

**Table 2.** Proposed and observed medication changes related to the patient’s prioritised health outcome

**Prioritised health outcome (n = 19)^a^**	**Type of proposed change**					**Total proposed changes**	**Total observed changes**
	**Stop**	**Dose increase**	**Dose decrease**	**Switch**	**Unknown**		
Remaining alive (*n* = 6)	5 Desloratadine Mebeverine^b^ Omeprazole Tamsulosin Tiotropium	1 Levothyroxine	4 Domperidone Macrogol (2×)^c^ Simvastatin	1 Temazepam to Melatonin	0	**11**	**3 stopped** Mebeverine^b^ Macrogol (2×)^c^
Maintaining independence (*n*= 7)	7 Alendronic acid^b^ Atorvastatin Hydrochlorothiazide Omeprazole Propranolol^b^ Simvastatin Tamsulosin	1 Salbutamol	2 Acetylsalicylic acid Allopurinol^c^	0	0	**10**	**3 stopped** Alendronic acid^b^ Propranolol^b^ Allopurinol^c^
Reducing pain (*n*= 1)	1 Citolapram	0	0	0	0	**1**	**0**
Reducing other symptoms (*n*= 5)	8 Alendronic acid^b^ Atorvastatin Calcium/vitamin D^b^ Ferrous sulphate^b^ Gliclazide^b^ Simvastatin (3×)^b^	0	0	2 Morphine to Buprenorphine Acenocoumarol to Acetyl-salicylic acid^c^	1 Morphine^c^	**11**	**7 stopped** Alendronic acid^b^ Calcium/vitamin D^b^ Ferrous sulphate^b^ Gliclazide^b^ Simvastatin (2×)^b^ Acenocoumarol^c^ **1 dose decrease** Morphine^c^
**Total**	**21^a^**	**2**	**6**	**3**	**1**	**33^a^**	**14**

a One patient gave the highest priority to both maintaining independence and reducing other symptoms, and is not included (proposed but not observed stop of betahistine).

b Observed change in agreement with the proposed change.

c Observed change adapted from the proposed change, or agreement unknown.

### Prioritised health outcome and medication changes

Table 2 shows the prioritised health outcome of the patients with the proposed and observed medication changes. For the six patients who prioritised remaining alive as the most important health outcome, 11 medication changes were proposed, mainly stopping (5 times) or dose decrease (4 times). Proposed changes involved mainly drugs for the alimentary tract (A02, A03, A06) and respiratory system (R03, R06). For three patients a medication stop was observed at follow-up, all for the alimentary tract (Table 2).

For the seven patients who gave maintaining independence the highest priority, 10 medication changes were proposed, mainly stopping (7 times) or lowering in dosage (2 times). This partly involved drugs for the cardiovascular system (C03, C07, C10), but also drugs from a range of other therapeutic subgroups (Table 2). For three patients a medication stop was observed at follow-up.

For the patient who prioritised reducing pain as most important, one medication stop was proposed but not observed.

For the five patients who gave reducing other symptoms the highest priority, 11 medication changes were proposed, mainly stopping (8 times) of preventive medication, including lipid-modifying agents and drugs for osteoporosis (Table 2). For three patients, seven medications were stopped and one was decreased in dose at follow-up.

## DISCUSSION

### Summary

GPs proposed 34 medication changes for 20 patients when using a health outcome prioritisation tool during medication review of 59 patients. At follow-up, 14 medication changes for 10 patients were observed, four of which were adaptations of the originally proposed changes. The proposed and observed changes involved mainly stopping of medication. More changes were observed for patients who prioritise ‘reducing other symptoms’ than for patients whose highest priority was ‘remaining alive’ or ‘maintaining independence’.

Overall, the GPs proposed medication changes that seemed partly in line with the patient’s prioritised health outcome. In patients with ‘remaining alive’ as the highest prioritised health outcome, GPs proposed to stop or decrease especially symptom-relieving medication, such as omeprazole, tamsulosin, or macrogol. Few of these proposed changes, however, were observed at follow-up, but the proposed dose decreases for macrogol resulted in medication stops. This suggests that a stepwise reduction of medication was followed and may be a good approach to stop symptom-relieving medication. In patients who prioritised ‘maintaining independence’ as most important, GPs proposed to stop various preventive medication, such as statins and antihypertensives. Few of these proposed changes were observed at follow-up, and one may question whether stopping preventive medication is in line with the patient’s prioritisation. GPs may have had difficulty in deciding which medication might be stopped when patients prioritise ‘maintaining independence’ as most important. In contrast, in patients who judged ‘reducing other symptoms’ as most important, such preventive medication was often stopped as proposed.

### Strengths and limitations

The GPs included in this study were mostly GP trainers and may thus be more experienced in consultations with older patients with complex care plans than the average GP. Also, because GPs were free to choose which patients they would invite for the OPT consultation, these patients may have been more open to participate in a medication review than the average older patient with multimorbidity. Data on the medication used before consultation and on proposed medication changes were reported by the GPs in a questionnaire, whereas data on medications at follow-up were extracted from the EHR. Although this ensured objective assessment of the prescribed medication changes at follow-up, this does not guarantee that the patient has used the medications. In the case of chronic medication, it indicates that the patient has requested a repeat prescription. However, the follow-up period varied and may have been too short for some patients to detect medication changes in repeat medications in the EHR. In other patients, medication changes may not have been detected because they were amended in the meantime.

### Comparison with existing literature

The method provided by Fried, as applied in the OPT, is the only approach developed to identify priorities of patients with multimorbidity.^7^ Previously, it was found that consultations in which an OPT was used led to a better understanding of the medication by patients, a deepening of the patient–doctor relationship, and a better insight of the GP in the patient’s views on illness and treatment.^9^ Therefore, health outcome prioritisation could support patients and GPs to change medication.^13^^,^^14^ Knowing more about patient priorities may especially support decision-making when guideline recommendations are conflicting. For both doctors and patients, a shared determination of health priorities is not common practice.^15^

This is the first study in which health outcome prioritisation was used by GPs during medication review. In total, 34 medication changes were proposed for the 59 patients (0.6 per patient). In comparison, a combined clinical medication review with a web-based pharmaceutical care plan conducted in the Netherlands resulted in 0.9 proposed care interventions per patient, half of which were not medication changes but involved additional monitoring and compliance management.^16^ In another study looking at medication reviews conducted by community pharmacists in the Netherlands, 1.0 proposed interventions to stop or adjust the dose of medication were observed per patient.^17^ These rates, however, will depend on the number of patients included, the severity of morbidity, and the appropriateness of their current medication. Of more interest is the finding that using a tool that supports patient involvement in medication review leads mainly to proposed medication stops and dose decreases, but not the start of new medication. This is in contrast to medication reviews conducted by community pharmacists, where relatively fewer stops and more switches were proposed, as well as additions of new medication.^17^

At follow-up, 42% of the proposed changes led to observed changes. This is comparable to the medication reviews conducted by Dutch community pharmacists, which showed 46% of implemented interventions.^17^ It thus seems that using a tool supporting patient involvement does not increase the implementation rate of proposed medication changes. On the other hand, the type of proposed changes influences the success rate for implementation. High percentages are found for stopping potentially harmful medication.^16^ In this study, however, many proposals were made to stop preventive and symptom-relieving medication that was not necessarily harmful. The study showed that such medication was indeed stopped in around half of the proposed cases. The authors can only speculate as to why medication appeared not to be stopped in the other cases. Uncertainty about the benefits and risks of discontinuing medication in older patients with multimorbidity might play a role.^13^^,^^18^^,^^19^ Patients may become afraid or reluctant to stop when they believe in some benefit of the medication.^13^^,^^14^^,^^20^ It is currently not clear whether such beliefs are consistent with health outcome prioritisation. Another reason why proposed medication changes did not result in observed medication changes could lie in the process of cessation. The GP and patient may have agreed to stop the medication, including a reconsideration of this decision after a specific period.^14^^,^^20^ This may have led to restarting the medication. Also, external influences, such as from a specialist or family member, may lead to a reconsideration of the proposed medication change.^20^

### Implications for research and practice

Using an OPT to support GPs in proposing medication changes in line with the patient’s priorities resulted mostly in medication stops and dose decreases. Medication changes may be easiest for patients who prioritise reducing other symptoms as most important. Future research is needed to determine whether patients benefit from a medication review with an OPT.
